# Remifentanil versus Fentanyl for Assisted Reproductive Technologies: Effect on Hemodynamic Recovery from Anesthesia and Outcome of ART Cycles 

**Published:** 2011-09-23

**Authors:** Mohammad Hossein Jarahzadeh, Robab Davar, Mohammad Reza Hajiesmaeili, Ahmad Entezari, Fatemeh Musavi

**Affiliations:** 1 Anesthesiology and Critical Care Department, Shahid Sadoughi University of Medical Sciences and Health Services, Yazd, Iran; 2 Obstetrics and Gynecology Department, Shahid Sadoughi University of Medical Sciences and Health Services, Yazd, Iran; 3 Anesthesiology and Critical Care Department, Tehran University of Medical Sciences and Health Services, Tehran, Iran; 4 Research and Clinical Center for Infertility, Shahid Sadoughi University of Medical Sciences and Health Services, Yazd, Iran

**Keywords:** Analgesia, Assisted Reproductive Technologies, Fentanyl, Remifentanil

## Abstract

**Background:**

We conducted this study to compare the outcome of assisted reproductive technology (ART) procedures and recovery from anesthesia in women who received opioid analgesia with remifentanil versus fentanyl.

**Materials and Methods:**

This double-blind, randomized clinical trial was carried out in the Yazd Research and Clinical Center for Infertility, Yazd, Iran. We studied 145 women who were participants in an ART program. During the first phase of the study, all patients underwent induction of anesthesia with thiopental and received analgesia with remifentanil or fentanyl. The primary endpoint was pregnancy rate per transfer. The numbers of oocytes collected, fertilized and cleaved were recorded, as was the number of oocytes transferred and recovery profile. In the second phase of the study, all patients were followed for outcome of ART cycle.

**Results:**

This study suggested that in women undergoing transvaginal ultrasound-guided oocyte retrieval procedures, the likelihood of a successful pregnancy was higher with a remifentanil-based monitored anesthesia care (MAC) technique than with a fentanyl-based MAC technique. The recovery from anesthesia was significantly better in the remifentanil group versus fentanyl group.

**Conclusion:**

The results of this study suggest that remifentanil in clinical practice is superior to fentanyl (Registeration Number: IRCT201009283468N3).

## Introduction

Controversy exists regarding the effects of anesthetic drugs administered during transvaginal puncture procedures for oocyte retrieval on ART outcome. Anesthetics have been detected in follicular fluid, (1–3); both animal (4) and human (5) studies suggest that these drugs may adversely affect oocyte fertilization and embryonic development. As a result, the optimal anesthetic technique for these assisted reproductive technology (ART) procedures is unknown.

Concerns regarding the potentially deleterious effects of anesthetic drugs have led to the use of anesthetic techniques that minimize exposure. Increasingly, these procedures are performed with sedative and/or analgesic drugs as part of a monitored anesthesia care (MAC) technique, particularly in oocyte retrieval (6).

Remifentanil, which is a rapid and ultra-short acting opioid analgesic, has been successfully used for ultrasonic-guided oocyte retrieval procedures as part of an MAC technique.(7, 8) We predict that the use of short acting anesthetic drugs is useful because shorter exposure for the oocyte has a lower adverse effect on oocyte quality and outcome of assisted reproductive cycles. Thus, we conducted this double-blind, randomized clinical trial study to compare the outcome of ART procedures in women who received opioid analgesia with remifentanil versus fentanyl for oocyte retrieval.

## Materials and Methods

This double-blind, randomized clinical trial was carried out in the Yazd Research and Clinical Center for Infertility, Yazd, Iran. A total of 145 American Society of Anesthesiologists (ASA) physical status I women who were participating in an intracytoplasmic sperm injection (ICSI) program were studied. The study was approved by the Institutional Review Board at the Yazd Research and Clinical Center for Infertility, Shahid Sadoughi University of Medical Science and Health Services. Written informed consent was obtained from all participants. All patients were scheduled for identical ovarian stimulation and ultrasonically guided transvaginal follicular aspiration protocols. Women underwent microinjection cycles with a long protocol.

During the study (March 2006 to January 2007), all patients received standardized monitored anesthesia care with remifentanil (1μg/kg IV) or fentanyl (2μg/kg IV) and thiopental (5 mg/kg IV) after 2 minutes. In the first phase of the study, we compared the mean systolic and diastolic blood pressure (millimeter Hg) in addition to the mean pulse rate (beats/minute).

If serum β human chorionic gonadotropin (HCG) level was greater than 10 after the 12^th^ week of gestation, the procedure was considered a success. The primary endpoint was pregnancy rate per transfer. The numbers of oocytes collected, fertilized and cleaved were recorded, as was the number of oocytes transferred and recovery profile. In the second phase of the study, all patients were followed for outcome of ART cycle.

Statistical analysis consisted of the chi-square test for nominal and student’s t test for numerical data using SPSS computer software (version 11.5), with p values <0.05 statistically significant.

Only mature MII oocytes were included in the ICSI program. After 16 to 18 post-oocyte microinjections, all oocytes were microscopically observed for signs of fertilization. Fertilization was confirmed when two pronuclei were present within the ooplasm. The rate of fertilization was calculated as the percentage of the fertilized oocytes per MII oocytes. Exactly 24 hours after fertilization, cleaved embryos were assessed and graded according to the degree of fragmentation and size of blastomeres. These were categorized into four groups: A (score 18-20), B (score 16-17), C (score 14-15) and D (score 12-13). In general, grade D embryos were discarded.

## Results

**Table 1:** Patient characteristics in the two study groups


Characteristics	Remifentanil (n=70)	Fentanyl (n=75)	P-value

**Age**	29.63	29.04	0.467
**Duration of infertility**	8.39	7.91	0.577
**Good oocytes (n)**	3.68 ± 3.44	3.04 ± 2.86	0.231
**Embryo(n)**	5.11 ± 3.48	3.58 ± 2.99	0.006
**Embryo Score**	17.62 ± 2.16	16.8 ± 2.29	0.032
**Transferred embryos (n)**	2.33 ± 1.21	2.26 ± 1.01	0.699
**Chemical Pregnancy (n)**	19	16	0.667
**Clinical Pregnancy (n)**	15	12	0.041


**Table 2:** Patient recovery profile in the two study groups


Characteristics	Remifentanil(n=70)	Fentanyl(n=75)	P-value

**Systolic blood pressure (mm Hg) **	12.84 ± 1.29	12.84 ± 1.67	0.423
**Diastolic blood pressure (mm Hg) **	7.55 ± 0.79	7.59 ± 0.81	0.723
**Heart rate (beats/minute)**	80.59 ± 7.49	84.56 ± 9.47	0.006


Although the numbers of oocytes harvested, fertilized and transferred in both study groups were similar, the pregnancy rate was significantly higher after remifentanil than after fentanyl (21.43% vs. 16%) (p<0.05). However, the recovery times were significantly shorter with the remifentanil group versus the fentanyl group (p< 0.05; Table 2).

## Discussion

This prospective study suggests that in women undergoing transvaginal ultrasound-guided oocyte retrieval procedures, the likelihood of a successful pregnancy is higher with a remifentanil-based MAC technique than with a fentanyl-based MAC technique.

Anesthetic drugs have been detected in follicular fluid, (1–3,^9^) and a longer period of exposure in the general anesthesia group may have enhanced the deleterious effects of these drugs on the oocyte and/or follicular structures, (10) thereby interfering with the reproductive process.

These findings are supported by a recent preliminary report by Toon et al. (11) suggesting an increased pregnancy rate in women having spinal compared with general anesthesia for oocyte retrieval.

Interestingly, the use of electroacupuncture in combination with a paracervical block for oocyte aspiration has been judged a good alternative to an opioid-based MAC technique, with an even higher pregnancy rate (12).

It is difficult to identify precisely which anesthetic drug was responsible for the difference in pregnancy outcome observed between the MAC and general anesthesia groups. Of interest, Stapleton et al. were unable to demonstrate a difference in outcome of *in vitro* fertilization (IVF) or ICSI procedures after changing from general anesthesia with propofol and alfentanil to an MAC technique involving the same anesthetic and analgesic drugs. Similarly, other investigators were unable to identify a negative impact of the use of propofol on ART outcome when comparing MAC sedation techniques with or without propofol, (13, 14) or general anesthesia with propofol to a local anesthetic (paracervical) block technique (12).

The duration of anesthesia or analgesia, as well as the procedural times, was significantly shorter with MAC than with general anesthesia. Interestingly, a similar finding has been reported recently in outpatients undergoing anorectal surgery procedures with MAC versus general anesthesia (15). It is certainly possible that the decrease in the time of drug “exposure” in the MAC group versus general anesthesia may have contributed to the improved pregnancy rate.

## Conclusion

Pregnancy rates in women undergoing transvaginal oocyte retrieval for ART were higher with a remifentanil-based MAC technique than with a fentanyl-based MAC.

## References

[1] Soussis I, Boyd O, Paraschos T, Duffy S, Bower S, Troughton P (1995). Follicular fluid levels of midazolam, fentanyl, and alfentanil during transvaginal oocyte retrieval. Fertil Steril.

[2] Endler GC, Stout M, Magyar DM, Hayes MF, Moghissi KS, Sacco AG (1987). Follicular fluid concentrations of thiopental and thiamylal during laparoscopy for oocyte retrieval. Fertil Steril.

[3] Achwal M, Abuzeid M, Bovenschen JL, Lawrence K, Jones ML, Verrill H (1999). Remifentanil and fentanyl concentrations in follicular fluid during transvaginal oocyte retrieval. Anesthesiology.

[4] Chetkowski RJ, Nass TE (1988). Isoflurane inhibits early mouse embryo development in vitro. Fertil Steril.

[5] Vincent RD Jr, Syrop CH, Van Voorhis BJ, Chestnut DH, Sparks AE, McGrath JM (1995). An evaluation of the effect of anesthetic technique on reproductive success after laparoscopic pronuclear stage transfer.Propofol/nitrous oxide versus isoflurane/nitrous oxide. Anesthesiology.

[6] Wilhelm W, Hammadeh ME, White PF, Georg T, Fleser R, Biedler A (2002). General anesthesia versus monitored anesthesia care with remifentanil for assisted reproductive technologies: effect on pregnancy rate. J Clin Anesth.

[7] Wilhelm W, Biedler A, Hammadeh ME, Fleser R, Grueness V (1999). Remifentanil for oocyte retrieval: a new singleagent monitored anaesthesia care technique. Anaesthesist.

[8] Casati A, Valentini G, Zangrillo A, Senatore R, Mello A, Airaghi B (1999). Anaesthesia for ultrasound guided oocyte retrieval: midazolam/remifentanil versus propofol/fentanyl regimens. Eur J Anaesthesiol.

[9] Hammadeh ME, Braemert B, Baltes S, Georg T, Rosenbaum P, Schmidt W (2000). Relationship between ovarian stimulation regimen and cytokine concentration in follicular fluid and their effect on fertilization and pregnancy outcome of patients undergoing ICSI program. Am J Reprod Immunol.

[10] Toon H, Kane P, Sweitzer B (2000). Is spinal anesthesia preferable to general anesthesia for oocyte retrieval?. Anesthesiology.

[11] Hein HA, Suit CT, Douning LK (1997). Effect of intravenous sedation on the outcome of transvaginal oocyte retrieval: a comparative study of propofol- and methohexital-based techniques. BUMC Proc.

[12] Maltepe F, Kocaayan E, Ugurlu BS, Akdeniz B, Guneri S (2006). Comparison of remifentanil and fentanyl in anaesthesia for elective cardioversion. Anaesth Intensive Care.

[13] Lacombe GF, Leake JL, Clokie CM, Haas DA (2006). Comparison of remifentanil with fentanyl for deep sedation in oral surgery. J Oral Maxillofac Surg.

[14] Guo X, Yi J, Ye T, Luo A, Huang Y, Ren H (2003). Comparison of remifentanil and fentanyl in patients undergoing modified radical mastectomy or total hysterectomy. Chin Med J (Engl).

[15] Beers RA, Calimlim JR, Uddoh E, Esposito BF, Camporesi EM (2000). A comparison of the cost-effectiveness of remifentanil versus fentanyl as an adjuvant to general anesthesia for outpatient gynecologic surgery. Anesth Analg.

